# Investigation of the Chemical Content and User Comments on Facial Cleansing Products

**DOI:** 10.7759/cureus.38673

**Published:** 2023-05-07

**Authors:** Semih Güder, Hüsna Güder

**Affiliations:** 1 Dermatology, Bezmialem Vakıf University, İstanbul, TUR; 2 Dermatology, Maltepe University Faculty of Medicine, İstanbul, TUR

**Keywords:** cleansing products, surfactants, fake cosmetics, facial cleansers, cosmetic ingredients

## Abstract

Background: The goal of skin cleansing is to reduce sebum and exogenous pollutants and control the skin microbiome. Surfactants in cleansers dissolve hydrophobic substances in an aqueous phase and allow them to move away from the skin's surface. The negative effect of surfactants on the skin barrier can be reduced by changing the solution properties. As a dermatologist in the group of patients we encounter in our clinical dermatology practice who recommends face wash products, we thought of conducting this research in order to determine the product contents to identify the products with the highest user satisfaction so that we can easily make the selection of the right product and direct the patients correctly.

Materials and methods: We planned to conduct cross-sectional research. Ten facial cleansing products sold on the most popular website that sells dermo-cosmetic products online in our country were selected. In the selection of the website, the criterion of having the most Internet traffic was sought. Internet traffic data was obtained from www.similarweb.com. The classification of the identified key ingredients according to their chemical properties was used on https://cosmeticanalysis.com. Reviews for each of the ten products in total were examined from the most recent date to the oldest.

Results: We detected 87 different chemicals in ten different products. These basically consisted of surfactants, emollients (moisturizers), emulsifiers (cleansers), buffering (denaturators), herbal ingredients-antioxidants, solvents, and moisturizers. A total of 30 different surfactants were identified as the main cleaning ingredient in the examined products. Counterfeit product reporting was especially high on expensive products. No correlation was found between the number of surfactants in the products and the positive effects, such as cleansing and acne reduction and increase, and the negative effects, such as dryness, redness, burning, and smoothing/softening (p>0.05). There was a negative correlation between the cleansing effect of the products and the improvement and worsening of acne (p<0.05, p<0.001, respectively).

Conclusion: The bottom line is that a good facial cleansing product doesn't have to contain a lot of chemicals and surfactants. It should be kept in mind that expensive products may be counterfeit and should question whether the product is original or not on the local product detection system from the barcode number.

## Introduction

This article was included in the 9th International Congress of Medicine and Health Sciences Research in Ankara, Turkey, on March 18-19, 2022, as an oral presentation.

Facial cleansing is necessary for personal hygiene. In addition, the appearance of the skin on the face is essential in terms of health and beauty. The history of soap, which is the predecessor of modern facial cleaning products, dates back to 2500 BC with the Sumerians. With the increase in cosmetic expectations other than health and hygiene, facial cleaning products are now offered in creams, gels, foams, bars, and liquids [1,2].

The goal of skin cleansing is to reduce sebum and exogenous pollutants and to control the skin microbiome. To maintain the skin barrier during cleaning, it is necessary to preserve endogenous lipids and the natural skin structure. The surfactants in the cleansers dissolve the hydrophobic substances in an aqueous phase, allowing them to move away from the skin’s surface [3].

The components of Skin cleansing products also have therapeutic benefits. For example, product ingredients improve disease symptoms and can reduce possible skin irritation caused by topical medications. In addition, they can cause changes in the superficial and deep layers of the epidermis and have undesirable effects such as irritation, allergy, and cytotoxicity [4].

Surfactants often cause irritant reactions similar to allergic reactions due to their ability to dissolve lipid membranes when they come into contact with the skin [5]. The North American Contact Dermatitis Group reported a prevalence of contact allergy due to a group of surfactants in hypoallergenic fluid scavengers between 0.9% and 2.3% [6]. No irritant reaction was observed in a study in which eight surfactants commonly used in skin cleansers were evaluated by patch testing on 105 patients. In contrast, only one surfactant had a sensitizing effect of around 5% [7].

The negative effect of highly active substances on the skin barrier can be reduced by changing the solution properties. For example, adding polymers, such as polyethylene oxide and polyethylene glycol, to the solution and adding amphoteric surfactants or glycerin minimizes the deterioration of the skin barrier [3].

People who need a facial cleansing product have many options to search for information and access therapies other than consulting a dermatologist for treatment recommendations. Therefore, encountering and retaining these patients can be difficult for dermatologists. Dermatologists need to provide satisfactory service because these patients can quickly seek treatment elsewhere via the Internet or other non-dermatologist options [8].

We conducted this research to determine the products with the highest user satisfaction and side effects reported by users. Although the research is based in Turkey, we believe that our results will reflect the general population because the products we reviewed are used worldwide.

## Materials and methods

Selection of products

We planned to select ten popular facial cleansing products sold on Turkey’s most popular website that sells dermo-cosmetic products online. In choosing the website, we sought the website with the most Internet traffic. Internet traffic data was obtained from www.similarweb.com. The most popular website was identified as www.trendyol.com, an online shopping marketplace where sellers market and sell their products. We performed a search by typing “facial cleansers” into the website’s search module. The listed products were arranged by the number of comments received, from the most comments to the least. The site’s filtering criteria determined the number of comments.

Determination of product ingredients

In the top ten products, 87 chemical ingredients were identified from the “contains” section in the product description or from the product descriptions on the manufacturer’s website. The classification on https://cosmeticanalysis.com was used to group the identified basic ingredients according to their chemical properties.

Evaluation of user comments

Reviews for the ten products were examined from the newest to oldest. Reviews with the keywords "shipping and packaging," "super," "gorgeous," "smells good," "definitely buy," "great," "very bad," "awful," and "don't buy" were eliminated. Counterfeit and original product notifications were obtained from the comments. Based on the comments that users shared, the information obtained by comparing the barcode number of the product from the product detection system was used to distinguish between original and counterfeit products. We terminated the study when we collected 100 comments for each product.

Exclusion criteria

Search results containing duplicate products, those with undetermined content, comedone cleaning kits, and cleaning brush products containing the word “cleanser” were excluded from the study.

Statistical analysis

SPSS Statistics version 25.0 (IBM Corp. Released 2017. IBM SPSS Statistics for Windows, Version 25.0. Armonk, NY: IBM Corp.) was used to analyze the variables. Quantitative variables were shown as n (%) in the tables. Pearson correlation analysis was used to examine the relationship between the number of surfactants in the products and the positive and negative effects reported by users. A p-value less than 0.05 was considered statistically significant.

## Results

We detected 87 chemicals in the ten products. These primarily consisted of surfactants, emollients (moisturizers), emulsifiers (cleansers), buffering (denaturants), smoothing agents, herbal ingredients (antioxidants), solvents (humidifiers), chelators (humidifiers), deodorizers, film-forming (antistatic), and perfumes. Apart from the substances used for buffering and preservative purposes, 69 of 87 chemicals were specific to the product (Table 1).

**Table 1:** Chemicals in product ingredients SLES: sodium laureth sulfate, SLS: sodium lauryl sulfate, PEG: polyethylene glycol

Product ingredient	Chemicals
Surfactants	Acrylates copolymer, carbomer, coco glucoside, cocamidopropyl betaine, cocamidopropyl hydroxysultaine, decyl glucoside, disodium cocoamphodiacetate, disodium cocoyl glutamate, hydroxy decanoic acid, laureth 2, lauric acid, PEG 6 caprylic capric glycerides, PEG 8, PEG 40 hydrogenated castor oil, PEG 60 hydrogenated castor oil, PEG 90 glyceryl isostearate, PEG 120 methyl glucose dioleate, PEG 150 pentaerythrityl tetrastearate, pentyleneglycol, propyleneglycol, sodium cocoamphoacetate, sodium laurate, sodium lauroyl lactylate, sodium lauroyl methyl isethionate, sodium lauryl sarkocynate, sodium methyl cocoyl taurate, sodium myreth sulfate, SLES, SLS, zinc laurate
Emollients (moisturizers)	Caprylyl glycol, cholesterol, glyceryl oleate, rhamnose, sodium hyaluronate
Emulsifiers (cleaners)	Coconut acid, myristic acid, polysorbate 20, polysorbate 80, potassium sorbate, stearic acid
Buffering (denaturators)	Potassium hydroxide, sodium hydroxide, smoothing agent, niacinamide
Herbal ingredients (antioxidants)	*Aloe barbadensis* leaf, *Camellia sinensis*, ceramide AP, ceramide EOP, ceramide NP, copper sulfate, *Cucumis sativus* juice, *Cucurbita pepo* seed oil, *Cryptomeria japonica* leaf extract, ethylhexylglycerin, fructooligosaccharides, *Ginkgo biloba* leaf extract, grapefruit water, *Melaleuca alternifolia* tea tree leaf oil, *Nelumbo nucifera* flower extract, *Oenothera biennis* flower extract, panthenol, *Pinus palustris* leaf extract, phytosphingosine, *Pueraria lobata* root extract, *Saccharomyces* ferment, *Salix alba* bark water, tocopheryl acetate, *Ulmus davidiana* root extract, *Vitis vinifera* fruit water, zinc pyrrolidone carboxylic acid
Solvents (humidifiers)	Alcohol denat, butylene glycol, decanediol, ethyl hexanediol, glycerin, hexylene glycol, methylglucet 20, methyl propanediol, PEG 200 hydrogenated glyceryl palmate, propanediol
Chelators (humidifiers)	Mannitol, sorbitol, xylitol
Deodorizers	Linalool, zinc gluconate
Film-forming (antistatics)	Polyquaternium 10
Perfumes	

A total of 30 different surfactant substances were identified as the primary cleansing ingredient in the examined products (Table 2).

**Table 2:** Surfactant content in products SLES: sodium laureth sulfate, SLS: sodium lauryl sulfate, PEG: polyethylene glycol

Product	Surfactant content
LA ROCHE POSAY EFFACLAR	SLES
	PEG 8
	Cocamidopropyl betaine
	PEG 120 methyl glucose dioleate
BIODERMA SEBIUM FOAMING GEL	Sodium cocoamphoacetate
	SLES
	PEG 90 glyceryl isostearate
	Laureth 2
	Propyleneglycol
GARNIER HYALURONIC ALOE GEL	SLS
	Coco glucoside
	Cocamidopropyl betaine
	Pentylene glycol
NIVEA AQUA SENSATION GEL	Cocamidopropyl betaine
	Sodium myreth sulfate
	SLS
	PEG 40 hydrogenated castor oil
COSRX SALICYLIC ACID DAILY GENTLE CLEANSER	Lauric acid
	Sodium methyl cocoyl taurate
	Cocamidopropyl betaine
	PEG 60 hydrogenated castor oil
NIVEA REFRESHING FACE WASH FOAM	Acrylates copolymer
	Disodium cocoyl glutamate
	PEG 40 hydrogenated castor oil
	Propylene glycol
CERAVE FOAMING CLEANSER	Cocamidopropyl hydroxysultaine
	Sodium lauroyl sarcosinate
	PEG 150 pentaerythrityl tetrastearate
	PEG 6 caprylic capric glycerides
	Sodium methyl cocoyl taurate
	Sodium lauroyl lactylate
	Carbomer
DIADERMINE MICELLAR FACIAL CLEANSING GEL	Cocamidopropyl betaine
	Sodium lauroyl methyl isethionate
	Propylene glycol
	Coco glucoside
	Lauric acid
	Sodium laurate
AVENE CLEANANCE CLEANSING GEL	Disodium cocoamphodiacetate
	Sodium lauroyl sarcosinate
NEUTROGENA ANTI-ACNE FACIAL CLEANSING GEL	Cocomido propyl hydroxy sultaine
	Cocamidopropyl hydroxysultaine
	Cocamidopropyl betaine
	PEG 120 methyl glucose dioleate

The product with the most comments from users received 33,357 reviews, and the product with the fewest comments received 2370 reviews. The rates of getting one-star and five-star reviews were about the same across all products. Counterfeit product reporting was especially high on expensive products (Table 3).

**Table 3:** Price, number of user reviews, and original and counterfeit product notice

Product	Counterfeit product	Original product	Price (USD)	Number of comments	One star	Five stars
LA ROCHE POSSAY EFFACLAR	8.00 (19%)	34.00 (81%)	9.34	33357.00	1484.00 (4.4%)	17352.00 (52%)
BIODERMA SEBIUM FOAMING GEL	8.00 (28.6%)	20.00 (71.4%)	9.12	16534.00	570.00 (3.4%)	8656.00 (52.4%)
GARNIER HYALURONIC ALOE GEL	.00	.00	2.19	5077.00	110.00 (2.2%)	2330.00 (45.9%)
NIVEA AQUA SENSATION GEL	.00	.00	2.04	3897.00	70.00 (1.8%)	1942.00 (49.8%)
COSRX SALICYLIC ACID DAILY GENTLE CLEANSER	.00	2.00 (100%)	9.34	3730.00	95.00 (2.5%)	1998.00 (53.6%)
NIVEA REFRESHING FACE WASH FOAM	1.00 (50%)	1.00 (50%)	1.31	3675.00	109.00 (3%)	1719.00 (46.8%)
CERAVE FOAMING CLEANSER	33.00 (67.3%)	16.00 (32.7%)	7.15	3428.00	177.00 (5.2%)	1665.00 (48.6)
DIADERMINE MICELLAR FACIAL CLEANSING GEL	.00	.00	1.75	2977.00	68.00 (2.3%)	1428.00 (48%)
AVENE CLEANANCE CLEANSING GEL	5.00 (26.3%)	14.00 (73.7%)	9.05	2635.00	36.00 (1.4%)	1397.00 (53%)
NEUTROGENA ANTI-ACNE FACIAL CLEANSING GEL	.00	2.00 (100%)	1.82	2370.00	86.00 (3.6%)	1011.00 (42.6%)

The positive comments of products were "reduced acne," "smoothed or softened skin," and "reduced oiliness." The negative comments were that it caused redness, itching, burning, or dryness, increased acne, or had no effect (Table 4).

**Table 4:** Negative and positive effects reported in user reviews

Product	Redness	Itching	Burning	Dryness	Induced acne	No effect	Total negative reviews	Reduced acne	Smoothness	Reduced oily skin	Reduced oily skin
LA ROCHE POSSAY EFFACLAR	2,00	.00	2.00	13.00	4.00	5.00	26	13.00	7.00	54.00	54.00
BIODERMA SEBIUM FOAMING GEL	2.00	1.00	1.00	7.00	10.00	.00	21	9.00	12.00	58.00	58.00
GARNIER HYALURONIC ALOE GEL	1.00	.00	.00	11.00	.00	.00	12	.00	26.00	62.00	62.00
NIVEA AQUA SENSATION GEL	1.00	.00	2.00	5.00	2.00	1.00	11	2.00	20.00	68.00	68.00
COSRX SALICYLIC ACID DAILY GENTLE CLEANSER	1.00	.00	.00	5.00	5.00	2.00	13	18.00	7.00	62.00	62.00
NIVEA REFRESHING FACE WASH FOAM	5.00	.00	1,00	5.00	3.00	5.00	19	1.00	24.00	56.00	56.00
CERAVE FOAMING CLEANSER	1.00	.00	.00	6.00	1.00	5.00	13	2.00	4.00	81.00	81.00
DIADERMINE MICELLAR FACIAL CLEANSING GEL	2.00	.00	1.00	1.00	3.00	1.00	8	4.00	15.00	73.00	73.00
AVENE CLEANANCE CLEANSING GEL	.00	.00	.00	12.00	.00	4.00	16	18.00	11.00	55.00	55.00
NEUTROGENA ANTI-ACNE FACIAL CLEANSING GEL	2.00	1,00	.00	7.00	30.00	7.00	47	33.00	10.00	10.00	10.00

**Figure 1:**
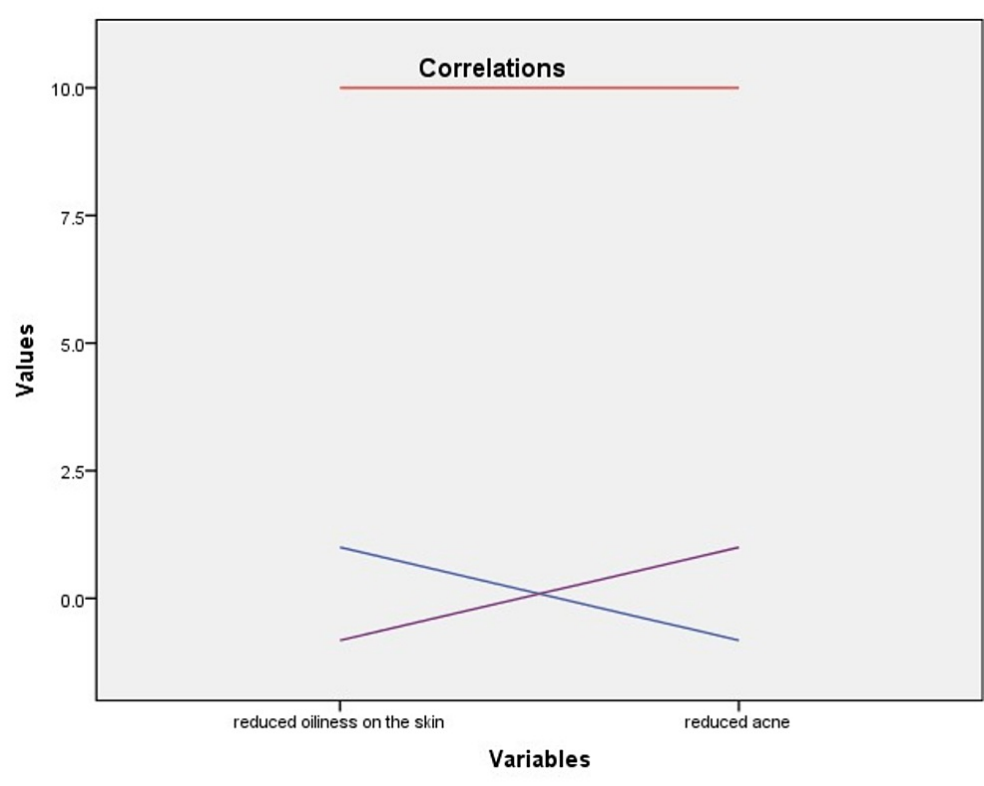
The relationship between cleansing effect and acne severity c: -0.822, p:0.002 (c:Pearson correlation coefficient)

There was a negative correlation between the cleansing effect of the products and the improvement of acne (p<0.05, Figure 1).

**Figure 2:**
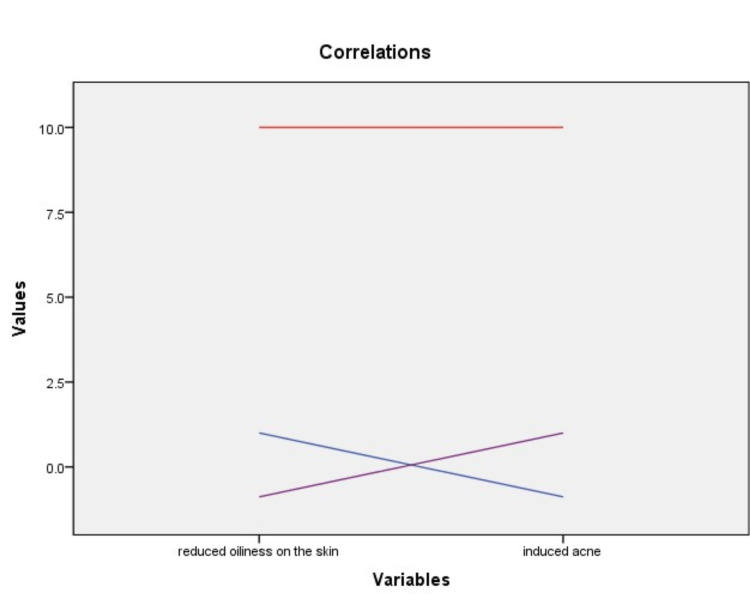
The relationship between cleansing effect and acne severity c: -0.881, p<0.001 (c:Pearson correlation coefficient)

At the same time, there was a negative correlation between the cleansing effect of the products and the worsening of acne (p<0.001, Figure 2).

No correlation was found between the number of surfactants in the products and the positive effects, such as cleansing and acne reduction or increase, and the negative effects, such as dryness, redness, and burning (p>0.05; Table 5).

**Table 5:** Correlations with surfactant number

		Redness	Itching	Burning	Dryness	Induced acne	Reduced acne
The number of surfactants	Pearson correlation	.135	.039	.020	-.568	-.038	-.411
	Sig. (two-tailed)	.709	.915	.956	.086	.916	.238
	N (10)						

## Discussion

The cosmetic facial cleansing products that our patients often consult us about and that we recommend have many variables. We conducted this research to determine the products with the highest user satisfaction and side effects reported by users.

We assumed that facial cleansing products would have more in common with each other, but we found that they contain many different chemicals. This may be due to manufacturers’ desires to make their products unique and not to imitate others.

The fact that the products have similar star ratings may be due to the adjustment of the sales site by the artificial intelligence algorithm. The high number of counterfeit product notifications for expensive products may be because those selling counterfeit products want to earn high profits via the original manufacturer brands. There is a risk of encountering counterfeit products when buying expensive facial cleansers online.

When we analyzed the reviews, we found a negative correlation between the cleansing effect and the improvement in acne. We hypothesized that this condition might be follicular irritant dermatitis due to irritants or allergens and that users may have interpreted this as increased acne [9]. There was also a negative correlation between the cleansing effect and the worsening of acne. We thought this might indicate that the product reduces acne [10].

We did not find a correlation between the number of surfactants and chemicals in the products and the cleansing effects, positive effects, and side effects. This may be because although the number of chemicals and surfactants in the products is few, the chemical content is in the right proportions or quantities. In addition, a product that contains a small number of surfactants or auxiliary chemicals does not necessarily have a low quality of cleaning.

Limitations of the study

The study’s main limitations are that we only selected ten of the most popular facial cleansing products and that the evaluation was subjective due to the electronic examination of user comments.

## Conclusions

We conclude that a good facial cleansing product does not need to contain many chemicals and surfactants. Users should also keep in mind that expensive products may be counterfeit and should query the local product detection system whether the product is original based on the barcode number. In order not to encounter counterfeit products, we recommend that they be purchased from a pharmacy instead of online shopping.
